# Evaluating a Trauma-Informed Care Training Program for Mental Health Clinicians

**DOI:** 10.1007/s40653-024-00639-0

**Published:** 2024-05-15

**Authors:** Shannon L. Stewart, Sarah Cloutier, Gabrielle King, Abigail Withers

**Affiliations:** Faculty of Education, University of Western, 1137 Western Rd, London, ON N6G 1G7 Canada

**Keywords:** Trauma-informed care, Training evaluation, Training program, Attitude toward TIC, interRAI

## Abstract

The aim of this study was to evaluate the interRAI Trauma-Informed Care (TIC) training program based on evidence-informed Collaborative Action Plans. Focus groups and the Attitude Related Trauma-Informed Care (ARTIC) questionnaire addressed clinicians’ and mental health professionals’ attitudes toward the application of TIC with their child and youth clients. An explanatory sequential design was conducted. In total, 105 clinicians and mental health professionals who participated in a 4-hour, in-person or virtual TIC training, two comprehensive seminars, and 28 trauma-informed training web-based modules completed the ARTIC questionnaire. Researchers conducted seven focus groups with clinicians/participants (*N* = 23) to discuss the views and effectiveness of the interRAI TIC educational training modules. To quantitatively measure the change of attitudes towards TIC, descriptive statistical analysis was completed using the means and standard deviation of the ARTIC scores at the initial time point, the follow-up time point, and the difference between scores at both time points. Paired sample t-tests were conducted on both the overall score and each of the subscales in each of the three samples (total sample, online subsample, and hybrid subsample). A thematic analysis was conducted to generate qualitative findings from the focus groups. Findings from the quantitative and qualitative analyses suggest that the interRAI TIC training provided clinicians with an improved sense of knowledge and ability to apply trauma-informed care planning with their clients.

## Introduction

In Canada, 32% of the adult population has experienced physical abuse, sexual abuse, and/or exposure to intimate partner violence during childhood (Afifi et al., 2014). These forms of childhood maltreatment can have a lasting impact on a child’s emotional and neurodevelopmental functioning (Oral et al., 2016) and often develop into mental health conditions, physical ailments, problems in school, interpersonal challenges, adjustment problems, behavioural and emotional difficulties, and substance abuse disorders (Afifi et al., 2014; Hertzman, 2013). Trauma interferes with an individual’s sense of safety, control, and self-regulation (Hertzman, 2013). To prevent such outcomes and minimize existing symptoms, experts suggest addressing issues related to trauma as early as possible, especially in cases of childhood trauma (Flynn et al., 2015).

Trauma informed care (TIC) has been widely accepted in various fields, including addiction and mental health, child and family services, and many educational activities and training programs. TIC is a critical, comprehensive, and systematic approach to patient care that brings forth the four R’s as core principles: (1) to *realize* the widespread impact of trauma; (2) to *recognize* the signs and symptoms of trauma not only in patients, but also their families and clinical team members; (3) to *respond* holistically by integrating knowledge about trauma; and (4) to actively resist *re-traumatization* (Huang et al., 2014). With proper training and program implementation, TIC has the potential to improve client engagement, care outcomes, practitioner well-being, and reduce service utilization and costs of care (Menschner & Maul, 2016). Incorporating a TIC lens is common in various fields and can help advise clinicians when to apply appropriate interventions and techniques to improve client outcomes (Richardson et al., 2012). In practices where a TIC lens is not utilized, there is a greater likelihood that clinicians will misunderstand their clients’ experiences (particularly when disruptive behaviour problems are present), which can weaken the quality of the therapeutic relationship (Richardson et al., 2012). Additionally, children are more likely to experience re-traumatization when healthcare workers who are uneducated in TIC use coercive practices that undermine patient autonomy, sense of safety, and self-esteem (Frueh et al., 2005; Muskett, 2014; Sweeney et al., 2018). As such, staff training in TIC is a critical component to better support children who have experienced trauma. Moreover, TIC training is especially critical as some mental health professionals have been found to exhibit unfavourable attitudes towards TIC practice and prefer to avoid exploring traumatic experiences (Amin et al., 2017; Sugg & Inui, 1992) or perceived trauma with their clients, either for fear of causing clients further distress or considering the practice to be outside their expertise (Palfrey et al., 2019).

To maximize the effectiveness of TIC approaches, the Public Health Agency of Canada (PHAC; Government of Canada, 2018) encourages TIC training for all organizational staff, not only employees who are clinicians. TIC training can involve teaching a combination of general principles as well as more specific interventions (Menschner & Maul, 2016), and research demonstrates that even a couple hours of training can result in significant changes. For instance, Liang and colleagues (2020) found that a 3-hour half day training program was just as effective as a 6-hour full-day program, and that schools that acquired ongoing TIC consultation, coupled with a training course, had the most positive attitudes toward TIC.

In recent years, there has been an increasing number of TIC initiatives implemented to promote trauma-informed practices in various settings including schools, health care, child welfare systems, and mental health organizations (i.e., Kenny et al., 2017; Kerns et al., 2016; Kuhn et al., 2019; Liang et al., 2020; Niimura et al., 2019; Palfrey et al., 2019). TIC programs are evaluated on various outcomes to determine their effectiveness. Although some studies evaluate client outcomes, typically studies focus on practitioner outcomes such as satisfaction with the program or changes in knowledge because of the TIC training program. For instance, in child welfare systems, after receiving TIC training, frontline staff reported a significant increase in use of TIC knowledge, practices, collaboration, skills in administering screening tools, and competency in identifying client needs and providing referrals (Lang et al., 2016; Kerns et al., 2016; Kuhn et al., 2019). For mental health service practitioners, a TIC workshop was rated relevant and useful to practice, which translated to a greater willingness to incorporate a trauma history in patient assessment practices (Palfrey et al., 2019). Participants reported an increase in their confidence, awareness and attitude towards the assessment and treatment of people exposed to trauma and adversity. There was a significant reduction in the worker’s perceptions of barriers (such as fear of causing further distress and lack of experience working with trauma; Palfrey et al., 2019). Although this research demonstrates that the development of TIC training programs is positively impacting staff outcomes, there is a lack of a standardized TIC training program that can be implemented across multidisciplinary sectors. Furthermore, there lacks a consistent tool to measure the effectiveness of new TIC training programs on staff outcomes. The variability in TIC training programs can lead to variability in the change in staff behaviour. Hence, research in this area is required to enhance and standardize TIC training programs and their evaluations to effectively reach the common goal of mitigating the impacts of trauma exposure.

### Measuring Attitudes and Knowledge of Trauma-Informed Care

A recently developed concept, Attitudes Towards Trauma Informed Care (ARTIC; Baker et al., 2016) can be used to capture TIC knowledge. The ARTIC questionnaire incorporates staff attitudes towards TIC, which are thought to drive the implementation and adaption of TIC behaviour, supporting the assessment of staff attitudes (Baker et al., 2016). Recent studies have used the ARTIC to examine staff understanding of TIC (e.g., Abudussatar, 2021; Berkout, 2018; Burkhardt, 2020; Daniels, 2021; Frerks, 2019; Jordan-Cox, 2018; Powers, 2020; Robertson et al., 2021; Smith, 2021). Of the few studies using the ARTIC-45 in their methodology, the majority have used it to measure and describe TIC attitudes at one time point. These studies included participants from different fields of work, including school staff (Abudussatar, 2021; Burkhardt, 2020; Daniels, 2021; Frerks, 2019; Powers, 2020; Robertson et al., 2021; Smith, 2021), childcare workers, direct care staff (Berkhout, 2018), and addiction counsellors (Jordan-Cox, 2018). Most studies used the ARTIC-45 to measure the relationship between TIC attitudes and another variable of interest, including classroom behaviour (Daniels, 2021), work experience (Powers, 2020), staff trauma levels (Berkhout, 2018; Jorden-Cox, 2018), and school staff characteristics (Robertson et al., 2021).

Researchers using the ARTIC-45 have separated participants using cut-off points to understand the participant population and set future goals to improve TIC attitudes. For example, Smith (2021) separated high school faculty into three groups based on the percentile rank of their ARTIC-45 scores; learn (0-25th percentile), grow (25th-75th percentile), or thrive (75th- 100th ). Similarly, Abdussatar (2021) used ARTIC-45 scores to split participants into three benchmark groups; low trauma-informed (means of 1–3), medium trauma-informed (means of 4–5) and high trauma-informed (means of 6–7). The ARTIC-45 has also effectively captured changes in TIC attitudes at two time points. For example, Orapallo and colleagues (2020, 2021) used a pre-post 1-year longitudinal design to measure overall changes in school staff attitudes towards TIC before and after the administration of a standardized TIC program.

The described studies support the ARTIC-45’s use as a tool to capture attitudes towards TIC. However, to date, no studies have assessed attitudes of multidisciplinary staff towards TIC. Despite the ARTIC-45’s ability to capture TIC attitudes of any staff providing care to individuals with trauma histories, participants of studies that used the ARTIC-45 have been limited to school staff (including teachers, principals, school counsellors, and administration staff), direct care staff, and clinicians in the field of substance use and addictions. Only one study has used the ARTIC-45 to capture attitudes towards TIC before and after implementing a TIC program (Orapallo, 2021), and consequently, more research is needed to support the use of the ARTIC-45 in this way. To date, we are not aware of any studies that have examined pre-post ARTIC-45 outcomes of a trauma-informed intervention designed for vulnerable clinically referred children and their families utilizing evidence-based care planning protocols.

### Current Study

We investigated the use of a standardized assessment-to-intervention approach being the interRAI Child and Youth Mental Health (ChYMH) Assessment (Stewart et al., 2015a, b) to support early identification and intervention in relation to trauma-informed care for children and youth. Integrated into the interRAI ChYMH is over 400 items and 30 evidence-informed care planning tools (Collaborative Action Plans; CAPs) to identify children and youth ages 4 to 18 years who are at heightened risk for specific care needs (e.g., attachment, interpersonal conflict, informal support; complete list of CAPs provided in Appendix A). Trained assessors gather information from multiple sources including the child/youth, their caregivers, teachers, clinicians, educators, and medical records. The interRAI ChYMH has robust psychometric properties (Lau et al., 2019, 2021; Li et al., 2021; Stewart & Hamza, 2017; Stewart et al., 2019a, b, 2021a, c, 2022a, b). The CAPs are triggered based on algorithms within the interRAI ChYMH that flag children with potential concerns in need of further clinical review. CAPs are designed to address these identified risk factors and clinical needs and provide strategies to equip clinicians with needed information to improve health outcomes for children, youth, and their families. Specifically, health promotion, prevention, and intervention practices to assist children and their families develop capacities to promote their overall health and wellness are integrated into the CAPs.

The current research seeks to investigate whether there are improved trauma-based beliefs regarding clinical implementation of best practices in domestic violence and abuse amongst clinicians and mental health professionals after completion of the interRAI TIC training program. The interRAI TIC training utilized a trauma-informed approach to training that was comprehensive and systematic and aligned with the four R’s as core principles (Huang et al., 2014). Since interRAI CAPs are evidence-informed care planning tools already incorporated into the interRAI ChYMH, trauma-informed care research and practices were integrated into each of the 30 CAPs. The first component of the training consisted of a 4-hour live training with a social worker and two 1-hour seminars on the *Trauma Informed Attachment* and *Trauma Informed Traumatic Life Events* interRAI CAPs. The second part of the training consisted of the remaining 28 CAPs in an online module format that took around eight hours for clinicians to complete.

It was anticipated that clinicians who participated in the interRAI TIC training would have a change in beliefs as measured by the ARTIC-45 (Baker et al., 2016) which could then result in clinical change to nurture the mental health and well-being of children exposed to specific types of interpersonal trauma (i.e., domestic violence, sexual, or physical abuse). The current study used a mixed methods approach to provide a novel contribution to the field and serve the current body of research supporting the use of the ARTIC-45 with multidisciplinary clinicians working in mental health agencies before and after receiving TIC training. Furthermore, the current study describes the development and implementation of a standardized TIC training program for multidisciplinary mental health organizations.

## Methods

### Sample

Trained clinicians and multidisciplinary mental health professionals were recruited from community agencies across Ontario, Canada to participate in the interRAI TIC training initiative. Mental health professionals had a range of years of experience in the field, various levels of education (e.g., college, undergraduate and graduate), and worked in different disciplines, such as psychology, social work, and child and youth work. Table 1 describes the demographic information collected from 68 mental health professionals who voluntarily provided demographic information. The demographic section of the study was considered optional and only those participants who volunteered and consented provided their demographic information.

**Table 1:** Descriptive statistics among samples

Characteristic	%, (*n*)		
	Online Subsample (*n* = 29)	Hybrid Subsample (*n* = 39)	Total Sample (*N* = 68)
Level of Education			
College or Bachelor	64.10 (25)	44.83 (13)	55.88 (38)
Master’s	35.90 (14)	55.17 (16)	44.12 (30)
Years of Experience			
0–7 Years	51.72 (15)	30.77 (12)	32.35 (22)
8 + Years	48.28 (14)	69.23 (27)	60.29 (41)
Discipline			
CY Worker	34.48 (10)	32.35 (22)	47.06 (32)
Social Worker	41.38 (12)	30.77 (12)	35.29 (24)
Other	24.14 (7)	12.82 (5)	17.65 (12)

**Fig. 1:**
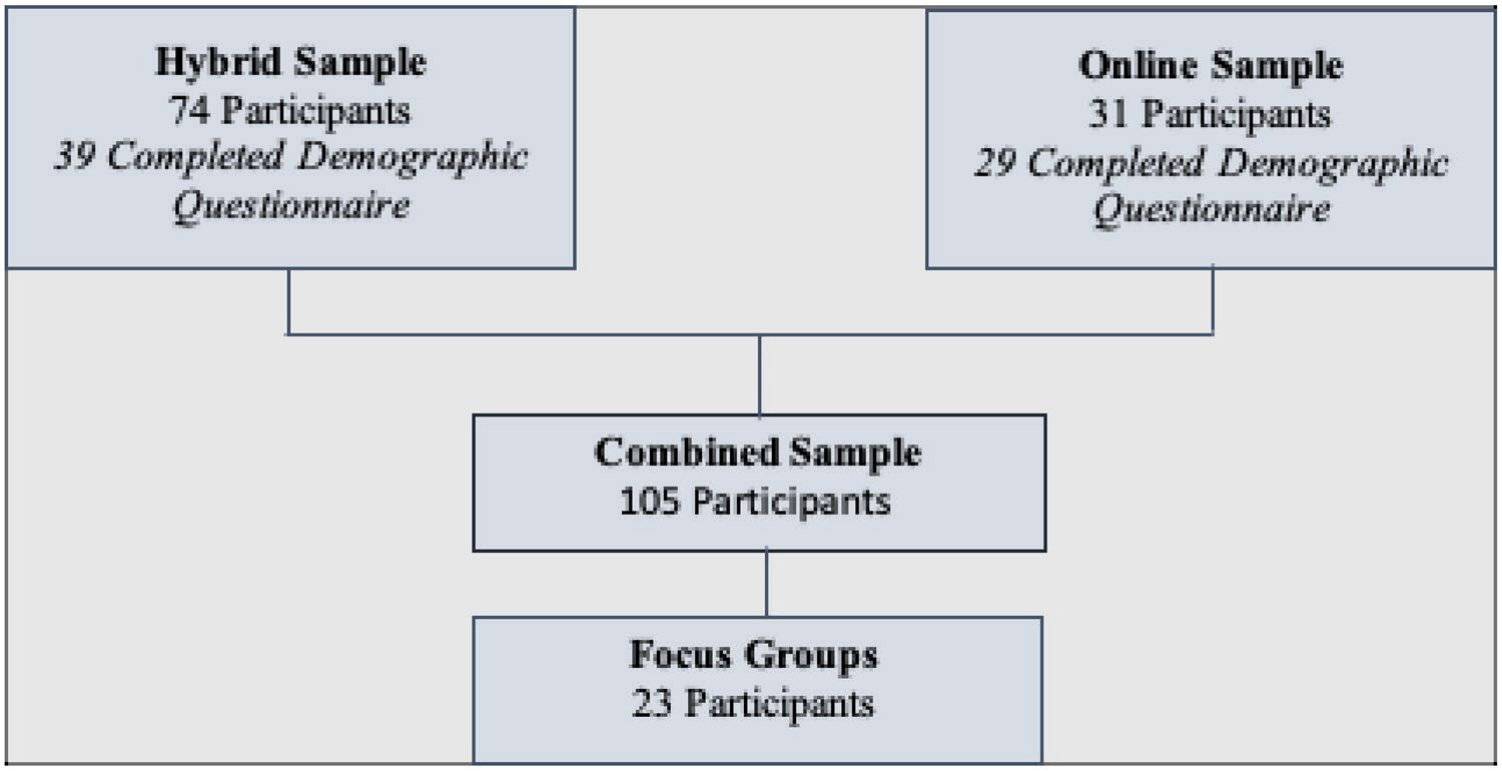
Sample groups. Breakdown of the sample groups. *Note.* All participants in the hybrid subsample and the online subsample completed pre and post ARTIC-45 questionnaires

One hundred and five participants (hereafter referred to as clinicians or participants) completed the interRAI TIC training program along with the pre and post ARTIC-45 questionnaire; these participants were included in the ‘total sample’. Due to COVID-19, of the one hundred and five participants, 74 participants completed training in a hybrid format, in which they were trained using a mixture of both in-person and online formats. These participants make up the ‘hybrid subsample’. Finally, 31 participants completed interRAI TIC training through a completely online format only, forming the ‘online subsample’. Please see Fig. 1 for a description of the samples.

Participants were deemed eligible for the focus groups if they completed five or more interRAI ChYMH assessments (to ensure each participant was familiar with the assessment and TIC modules) and were then recruited to participate. There were 44 participants were eligible to participate, and 23 participants consented to engage in the focus groups. Each focus group included participants employed from the same mental health agency and five mental health agencies were involved. Focus group participants completed their training either in an online only format or with two modules in-person and the remaining modules online. The participants included seven clinicians, five therapists, eight child and youth workers, and three participants whose disciplines cannot be disclosed for confidentiality purposes. Descriptive statistics among sample groups are listed in Table 1.

### Measures

#### Demographic Questionnaire

The demographic questionnaires consisted of four self-report questions, including the clinician’s level of education (e.g., college, bachelors, masters), their discipline (e.g., social work, child and youth worker), the number of years of clinical experience, and their comfort handling the trauma histories of others rated on a scale of 1–5 (1 = extremely comfortable and 5 = not at all comfortable).

#### Attitude Related to Trauma-Informed Care Scale (ARTIC)

The ARTIC-45 (Baker et al., 2016) scale is a validated measure containing 45 bipolar Likert scale items aimed to measure the respondent’s attitudes towards (TIC). Respondents must rate themselves on a 7-point spectrum between two items, one which is favourable to TIC and one which is unfavourable to TIC. To measure favourable attitudes toward TIC, items such as “The trauma-informed care approach is effective” were used whereas other items targeted unfavourable attitudes toward TIC: “The trauma-informed care approach is not effective”. Items were scored between 1 and 7 to represent their personal beliefs about their job in the past 2 months. Once certain items were reverse scored, a total scale score and subscale scores were calculated. There were five core subscales that evaluated attitudes toward TIC implementation: (a) Underlying cause of problem behaviour and symptoms; (b) responses to problem behaviour and symptoms; (c) on-the-job behavior; (d) self-efficacy at work; (e) reactions to work. The ARTIC-45 includes two supplementary scales that measure support for systematic TIC adoption: (f) personal support of TIC; and (g) system-wide support for TIC. Participants must complete most of the scale items (at least 2 of the 5 items within the supplementary subscale, and 4 of 7 items completed within the main subscales) for the main subscales. Higher scores represented more favourable TIC attitudes (Baker et al., 2021). The psychometric properties of the ARTIC have been validated in samples of education, human services, and health care staff (Baker et al., 2016). Findings indicated excellent internal consistency (a = .93), good test-retest reliability (*r* = .84 at ≤ 120 days, *r* = .80 at ≤ 121–150 days and *r* = .76 at 151–180 days), and good construct and criterion-related validity.

### Procedure

Ethics approval for the study was received. Clinicians were recruited from community agencies across Ontario, Canada through a verbal script and letter of information/consent to participate. A member of the research team met with participants virtually to review consent and confirm participation in the study. Participants were then given access to the online training modules for the interRAI ChYMH CAPs. Before starting the training, participants were asked to complete a demographic questionnaire to gather information about their experience and education (68 of 105 participants completed the questionnaire). All participants completed an initial ARTIC-45 questionnaire prior to starting the training.

#### interRAI TIC Training

Prior to the COVID-19 pandemic, the interRAI TIC training was provided both in-person and online. Participants first completed a 4 h, in-person training that was held during normal working hours by a seasoned social worker with expertise in TIC. Clinicians then participated in two comprehensive seminars: *Trauma Informed Attachment* and *the Trauma Informed Traumatic Life Events* from the *interRAI ChYMH Collaborative Action Plans* (Stewart et al., 2015a, b). Thereafter, participants completed an in-person training feedback form that evaluated training delivery in domains such as training length, specific content, or delivery method. The second component of the training was provided online using the project website. The website included publicly accessible information about the research project and team members. Participants were provided with a username and were directed to create a personal password to enter and access the remaining content on the website. The content included a general overview of the remaining 28 trauma-informed training modules based on the interRAI ChYMH Collaborative Action Plans (CAP). Each CAP had its own page, complete learning objectives, descriptions, and an embedded video (ranging from 8 to 61 min depending on topic complexity) and Portable Document Format (PDF) transcript that provided trauma-informed guidelines as well as the clinical application of the CAP. Once the training of the CAP was completed, five knowledge-testing true/false and multiple-choice questions were provided for each CAP. The participants must have correctly answered all five questions to successfully complete the module. Once all 28 CAPs were reviewed and the knowledge-testing questions were successfully completed, the participants received a certificate of completion. The participants were then asked to complete a post-ARTIC-45 questionnaire to help determine if the training altered the participants’ belief related to TIC.

During the COVID-19 pandemic, the initial in-person portion of the trauma-informed training was replaced with a virtual, live format. Thereafter, these same training seminars were video recorded and provided as online educational videos. Both the Trauma-informed Attachment video module and the Traumatic Life Events CAP videos were approximately one hour in length. The former was divided into 6 video segments while the latter was divided into 5 video segments. Similar to the remaining 28 CAP modules that were video-recorded prior to COVID-19, a PDF of the transcript of the video, a video presentation and knowledge-testing questions based on the CAPs were developed and integrated into the project website. On average, it took approximately 8 h to complete the 30 learning modules and knowledge-based testing questions, varying across participants depending on their learning style and approach.

#### Focus Group

To qualitatively receive feedback relating to the interRAI TIC training, clinicians who were participating in the project, completed 5 or more interRAI ChYMH, and fully completed the interRAI TIC training modules were invited to participate in focus groups. Those who volunteered by responding and providing consent for their participation in the focus group were involved. The focus groups were designed to allow for an intensive and authentic investigation into the participants’ views of the interRAI TIC training in relation to their beliefs about implementing TIC with their clients. The focus groups were structured so that participants within the same agency were involved in the session, rather than across agencies, to prevent any possible breach of private information shared across agency participants. Participants were asked questions about their experience completing the TIC training and shared their experiences, thoughts, feedback, and applications of the training within their role (see Appendix B for the questions). Focus groups were conducted virtually on zoom by members of the research team and lasted approximately 30 min. A total of seven interRAI TIC training focus groups took place between January and March 2022.

To mitigate potential bias during the focus groups, clinicians were asked non-leading questions (e.g., “to what degree, if at all…”). Different sessions were conducted for clinicians and managers to help clinicians feel more open and comfortable to share their feedback. All agencies that participated in the training were given the opportunity to participate in the focus group, with only those that voluntarily consented participated in the focus groups. To mitigate researcher bias, two members of the research team reviewed the transcripts and conducted the thematic analysis to generate the focus group themes, addressing any discrepancies via consensus.

### Analysis

For the descriptive analysis, only participants who had completed the demographic questionnaire as well as both the initial and the follow-up ARTIC questionnaire, were included in the sample. To examine trends in the responses to the questionnaire, a frequency analysis was conducted for each of the 3 questions (discipline, level of education, and years of experience) for each of the three sample groups.

For the inferential analysis, the data from participants who did not complete most of the subscale items or scales, were removed from the data set. To measure the change of attitudes towards TIC, the means, and standard deviations of the ARTIC scores at the initial time point and the follow-up time point were compared. A series of paired sample t-tests were used for the overall score and each of the subscales for the 3 samples (total sample, online subsample, and hybrid subsample). The assumption of normality was not satisfied for some subscales. As a result, the bootstrapping method was applied to estimate the results of the population that the sample was drawn from. For consistency, this method was applied to each test. All quantitative analyses were conducted using statistical package for social sciences (SPSS) software.

To analyze qualitative data from the focus groups, a thematic analysis was employed to systematically identify and organize patterns of meaning in the data to generate a shared understanding of the experiences relevant to the research questions (Braun & Clarke, 2021). Two members of the research team employed a six-phase approach to the thematic analysis as detailed by Braun and Clarke (2012). Researchers engaged in a dialogic process while coding to better understand and analyze the data. After all focus groups were complete and transcribed, researchers familiarized themselves with the data, discussed any patterns in the transcripts, and generated initial codes. Shared dialogue amongst researchers resulted in recoding and collapsing the codes into broader themes. Researchers engaged in a recursive process of returning to the data, conducting multiple rounds of analysis, and drawing thematic maps to reach a consensus on the themes. As the themes and subthemes were defined, researchers continued with the reiterative process of returning to the data to gather quotes and examples resulting in three overall themes.

## Results

### Quantitative Effectiveness of Training in Total Sample and Online and Hybrid Subsamples

A series of paired sample t-tests were conducted to examine the difference in scores between the initial and follow-up ARTIC questionnaires because of the interRAI TIC training in the total sample, online subsample, and hybrid subsample (see Table 2). A statistically significant improvement in attitudes towards TIC was found between the initial and the follow-up ARTIC total scores, after the interRAI TIC training, in the total sample (initial, *M* = 5.87, *SD* = .49; follow-up, *M* = 6.04, *SD* = .62; [*t*(104) = − 4.37, *p* = < .001*]), online subsample (initial, *M* = 5.90, *SD* = .52, follow-up; *M* = 6.22, *SD* = .53; [*t*(30)= -5.50, *p* = < .001*]) and hybrid subsample (initial, *M* = 5.85, *SD* = .48; follow-up, *M* = 5.96, *SD* = .64; [*t*(73) = − 2.22, *p* = .030*]). The breakdown per subscale is presented below.

**Table 2:** Initial and follow-up ARTIC score results in total sample and online & hybrid subsamples

Scale/Subscale Sample	*n*	Mean (SD)		Mean Difference (95% CI)	*P*-value
		Initial	Follow Up		
Total Score					
Total Sample	105	5.87 (0.49)	6.04 (0.62)	0.17 (-0.25, − 0.09)	< 0.001**
Online Subsample	31	5.90 (0.52)	6.22 (0.53)	0.32 (0.21, − 0.44)	< 0.001**
Hybrid Subsample	74	5.85 (0.48)	5.96 (0.64)	0.11 (-0.20, − 0.01)	0.030*
Underlying Causes of Problem Behavior and Symptoms					
Total Sample	93	5.73 (0.58)	5.95 (0.67)	0.22 (-0.31, 0.12)	< 0.001**
Online Subsample	25	5.91 (0.48)	6.16 (0.47)	0.25 (-0.42, − 0.08)	0.009*
Hybrid Subsample	68	5.66 (0.61)	5.87 (0.71)	0.21 (-0.33, − 0.09)	0.002*
Responses to Problem Behavior and Symptoms					
Total Sample	105	5.98 (0.66)	6.26 (.66)	0.28 (-0.37, − 0.19)	< 0.001**
Online Subsample	31	6.05 (0.55)	6.30 (0.53)	0.25 (-0.36, − 0.13)	< 0.001**
Hybrid Subsample	74	6.21 (0.52)	6.36 (0.56)	0.14 (-0.25, − 0.04)	< 0.001**
On the Job Behaviour					
Total Sample	105	6.21 (0.51)	6.39 (0.52)	0.18 (-0.27, − 0.09)	< 0.001**
Online Subsample	31	6.19 (0.50)	6.46 (0.40)	27 (-0.43, − 0.10)	0.003*
Hybrid Subsample	74	5.87 (0.63)	6.00 (0.78)	0.14 (-0.25, − 0.04)	0.018
Self-Efficacy at Work					
Total Sample	105	5.86 (0.63)	6.02 (0.73)	0.16 (-0.27, − 0.04)	0.011*
Online Subsample	31	5.83 (0.65)	6.08 (0.63)	0.25 (-0.45, − 0.05)	0.021*
Hybrid Subsample	74	5.87 (0.63)	6.00 (0.78)	0.13 (-0.25, − 0.01)	0.091
Reactions to the Work					
Total Sample	105	5.87 (0.63)	5.88 (.74)	0.01 (-0.13, 0.11)	0.882
Online Subsample	30	5.87 (0.68)	6.24 (0.60)	0.37 (-0.61, − 0.14)	0.006*
Hybrid Subsample	74	5.87 (0.62)	5.73 (0.75)	− 0.14 (-0.02, − 0.28)	0.053
Personal Support of Trauma-Informed Care					
Total Sample	98	5.54 (0.93)	5.72 (1.05)	0.14 (-0.32, 0.03)	0.120
Online Subsample	30	5.87 (0.91)	6.32 (0.78)	0.45 (-0.71, − 0.20)	0.007*
Hybrid Subsample	68	5.69 (0.86)	5.70 (0.93)	0.01 (-0.23, − 0.22)	0.958
System-Wide Support for Trauma-Informed Care					
Total Sample	98	5.74 (0.87)	5.89 (0.93)	0.18 (-0.39, − 0.02)	0.087
Online Subsample	30	5.66 (1.12)	6.08 (0.84)	0.42 (-0.70, − 0.15)	0.009*
Hybrid Subsample	68	5.50 (0.83)	5.56 (1.09)	0.07 (0.34, − 0.20)	0.587

*p< .05; **p< .001

#### Underlying Causes of Problem Behavior and Symptoms

The results from the initial and follow-up questionnaire indicate that the interRAI TIC training program resulted in a significant improvement in scores on the *Underlying Causes of Problem Behavior and Symptoms* subscale in the total sample (initial, *M* = 5.73, *SD* = .58; follow-up, *M* = 5.95, *SD* = .67; [*t*(92) = − 4.48, *p* = < .001**]), online subsample (initial, *M* = 5.91, *SD* = .48, follow-up; *M* = 6.16, *SD* = .52; [*t*(24)= -2.61, *p* = .009*]) and hybrid subsample (initial, *M* = 5.66, *SD* = .61; follow-up, *M* = 5.87, *SD* = .71; [*t*(67)= -3.51, *p* = .002*]).

#### Responses to Problem Behavior and Symptoms

The results from the initial and follow-up questionnaire indicate that the interRAI TIC training program resulted in a significant improvement in scores on the *Responses to Problem Behavior and Symptoms s*ubscale in the total sample (initial, *M* = 5.98, *SD* = .66; follow-up, *M* = 6.26, *SD* = .67; [*t*(104) = − 6.67, *p* = < .001**]), online subsample (initial, *M* = 6.05, *SD* = .55, follow-up; *M* = 6.30, *SD* = .53; [*t*(30)= -4.15, *p* = < .001*]) and hybrid subsample (initial, *M* = 5.94, *SD* = .70, follow-up; *M* = 6.24, *SD* = .71; [*t*(73)= -5.41, *p* = < .001**]).

#### On-the-Job Behaviour

The results from the initial and follow-up questionnaire indicate that the interRAI TIC training program resulted in a significant improvement in scores on the *On-the-Job Behaviour* subscale in the total sample (initial, *M* = 6.21, *SD* = .51; follow-up, *M* = 6.39, *SD* = .52; [*t*(104)= -3.79, *p* = < .001**]), online subsample (initial, *M* = 6.19, *SD* = .50, follow-up; *M* = 6.46, *SD* = .40; [*t*(30) = − 3.40, *p* = .003*]) and hybrid subsample (initial, *M* = 6.21, *SD* = .52, follow-up; *M* = 6.36, *SD* = .56; [*t*(73)= -2.43, *p* = < .018*]).

#### Self-Efficacy at Work

The results from the initial and follow-up questionnaire indicate that the interRAI TIC training program resulted in a significant improvement in scores on the *Self-Efficacy at Work* subscale in the total sample (initial, *M* = 5.86, *SD* = .63; follow-up, *M* = 6.02, *SD* = .73; [*t*(104)= -2.77, *p* = < .011*]) and the online subsample (initial, *M* = 5.83, *SD* = .65; follow-up, *M* = 6.08, *SD* = .63; [*t*(30)= -2.54, *p* = .021*]). There was no significant change in scores found between initial and follow-up questionnaire in the hybrid subsample.

#### Reactions to the Work

The results from the initial and follow-up questionnaire indicate that the interRAI TIC training program resulted in a significant improvement in scores on the *Reactions to the Work* subscale in the online subsample (initial, *M* = 5.87, *SD* = .68; follow-up, *M* = 6.24, *SD* = .60; [*t*(30)= -3.28, *p* = .006*]). However, there was no significant change in scores found between initial and follow-up questionnaire in either the total sample or the hybrid subsample.

#### Personal Support of Trauma-Informed Care

The results from the initial and follow-up questionnaire indicate that the interRAI TIC training program resulted in a significant improvement in scores on the *Person Support of Trauma-Informed Care* subscale in the online subsample (initial, *M* = 5.87, *SD* = .91; follow-up, *M* = 6.32, *SD* = .78; [*t*(29)= -3.62, *p* = .007*]). However, there was no significant change in scores found between initial and follow-up questionnaire in either the total or the hybrid subsample.

#### System-Wide Support for Trauma-Informed Care

The results from the initial and follow-up questionnaire indicate that the interRAI TIC training program resulted in a significant improvement in scores on the *System-Wide Support for Trauma-Informed Care* subscale in the online subsample (initial, *M* = 5.66, *SD* = 1.11; follow-up, *M* = 6.08, *SD* = .84; [*t*(29)= -3.08, *p* = .009*]). However, there was no significant change in scores found between initial and follow-up questionnaire in either the total or the hybrid subsample.

### Qualitative Results

Three overarching themes were the result of the thematic analysis of the focus group participants’ experiences, thoughts, and application of the interRAI TIC training. Overall, participants reported that the training enhanced their application of trauma-informed care with clients, facilitated integration of TIC into the fabric of their agency, and improved their understanding of the ChYMH and CAPs. A discussion of each theme is provided below.

#### Theme 1. The TIC Training Enhanced and Validated the Application of the Trauma-Informed Care Approach with Children, Youth, and Their Guardians

The training provided clinicians with the opportunity to enhance and apply their trauma-informed care knowledge and skills with their clients. Although many clinicians had previous TIC training and education, the training provided “good reminders” (Participant 10 and 18) and “refreshers” (Participant 6, 10, 16 and 18) regarding current evidence in the field. Ultimately, many clinicians viewed their client-care as more “thorough” (Participant 5, 6, and 15), “concrete” (Participant 5, 6, and 9) and “detailed” (Participant 6) and felt an increased sense of confidence because of the training. For instance, Participant 2 said:

I also feel more confident to talk about the concepts. For me, it also keeps it prevalent and at kind of the forefront of my work. (….) I’m meeting these needs through these potential triggered CAPs by implementing these specific pieces. So, I think it’s (…) that pathway that I forget often. And (…) this training has helped me reaffirm those pathways and in that I become more confident through my work in terms of having those conversations and providing strategies that connect with each other in a way that’s meaningful and goal-related to where the families are at.

Clinicians expressed that the TIC knowledge they received in the training was helpful for supporting caregivers and families. Clinicians applied their knowledge and informed families “that it was a specific trauma-informed based strategy that we can implement” (Participant 2). Clinicians noticed caregivers were “more engaged in the process and…in the dialogue” (Participant 6) when presenting the client’s assessments or follow-up assessments. For example, Participant 4 noticed guardians would “start to use or catch on to the language” so clinicians would provide them with the definition for a deeper understanding. Clinicians spoke about supporting guardians by providing them with resources from the online training platform to help “put those into perspective in their own homes, working with their own children” (Participant 5). Therefore, because of the training, clinicians gained the resources and tools to enhance their application of TIC and the caregiver’s understanding of their child’s treatment.

Although many clinicians were experienced and/or educated in TIC, the training provided them with reminders of things they talk about daily and further “validated” (Participant 2, 11, and 12) their current work. For instance, Participant 2 said: “I’m doing more psychoeducation and family support and really getting families to understand that some of these behavioural pieces stem from traumatic experiences. It’s been validating that the things that I’m doing with families are trauma informed care related”. The training validated the work clinicians do with their clients, and the reasoning or “why” (Participant 2, 8, 9 and 16) behind it. This validation has “streamlined” (Participant 2) their work and their reasoning for implementing TIC practices.

Clinicians also reported an increased use of client-focused, TIC language as the training provided relevant and easy-to-understand TIC definitions, terms, and concepts. The training helped clinicians communicate with their clients and know “how to relay the language to youth and describe it in a certain way that they were able to understand what the language was” (Participant 7). Clinicians also noted that the training encouraged an empathetic approach to address and validate their client’s traumatic experience, resulting in more youth-engagement in their treatment. For example, Participant 1 said: “I’ve had some discussions with youth about that and (…) they felt really heard and understood (…) I think with the trauma-informed lens you can get more engagement with the youth”.

Clinicians discussed that since completing the training, they were able to develop a shared understanding and verbiage of TIC language. Clinicians reported “hearing a lot of the [TIC] language” (Participant 11) in their meetings and regular communications. They identified more open and streamlined communication amongst staff because they “feel safe in that setting” (Participant 16) to have conversations “in a more knowledgeable way” (Participant 16). This shared understanding of the language and knowledge was notably helpful for newer staff engaging with more seasoned staff. For instance, Participant 6 said:

We talked about the increased communication around using the appropriate language. Being able to use the appropriate language is helpful for newer or less seasoned staff. It gives them a better understanding right off the top, and it promotes the use of the language. So, I have found that very effective.

Furthermore, the TIC training improved the application of TIC treatment planning amongst teams of clinicians working with the same client to enhance case management. As a result of the shared TIC knowledge, clinicians were integrating the TIC language and interRAI ChYMH CAPs into their documentation, assessments, and reports. For example, Participant 5 noted that “having a baseline where we can all relate to and the concepts being consistent throughout our three live-in treatment sites, it is making it a lot easier in team meetings, in plan of cares, in debriefs”. In staff meetings and discussions, clinicians applied the TIC language to treatment planning as it “brings us back to that trauma-informed focus” (Participant 7) and helps develop treatment goals to “tie it all together (…) develop the plan (…) [and] get the goal going” (Participant 8). Clinicians found the training provided further opportunity to hear new perspectives about TIC concepts and information from fellow clinicians. Clinicians were more comfortable and confident explaining to fellow clinicians that they were “implementing this because it’s a trauma-based strategy that the family would benefit from at this point in time” (Participant 2).

#### Theme 2. The Completion of the TIC Training Helped Integrate TIC Into the Fabric of the Agency and Provide Timely Services

Clinicians expressed that because of the TIC training, their agency “leadership supports the time that it takes to implement those concepts on a one-to-one level with families” (Participant 2). Clinicians were also better equipped to justify the amount of time it took for various tasks with each client as they were provided with knowledge of “approaches that are shown to be really effective and trauma informed” (Participant 9) to justify their work.

Furthermore, the completion of the TIC training gave clinicians the knowledge and skills to advocate for children and youth to receive earlier services within their agency. For instance, a clinician noted that “if we can identify this early on then we can certainly advocate for earlier services” (Participant 11). Clinicians reported the modules enhanced their identification of families that would benefit from treatment rooted in a TIC approach. The modules also provided strategic approaches to service utilization to ensure that clients received the appropriate treatments needed for domestic violence and abuse. For instance, Participant 3 stated:

There’s CAPs coming up that are identifying trauma, and that helps to determine, if it would be helpful to bring me in to help support either a parent or a youth or do some family work together? …. Instead of waiting for the waitlists and the community it is helpful if I can do some of that pre-work until they can get in to do that longer term work.

Many clinicians identified situations to impact the wait times experienced by clients exposed to trauma or domestic violence and abuse. For example, clinicians felt the training increased their awareness of trauma in their clients and improved their early identification of these concerns to help with the timeliness of services provided to clients.

#### Theme 3. The TIC Training Provided Clinicians a Deeper Clinical Understanding and Utility of the interRAI ChYMH Assessment and CAPs

Clinicians saw improvements in their understanding and utility of the interRAI ChYMH after completing the TIC training. For example, clinicians developed a “better and more comprehensive understanding” of the “specifics of the interRAI ChYMH” (Participant 6) to “implement the ChYMH into my own treatment” and use it as a “guidepost along the way” (Participant 13). Additionally, the training prompted more conversations about the interRAI ChYMH amongst staff members as they could relate to the knowledge and language being used. One clinician stated, “I have a better understanding of the language they are using and understanding the CAPs and (…) how the interRAI and ChYMH works” (Participant 4).

Moreover, the completion of the TIC training enhanced clinicians’ understanding and utility of the specific CAPs. For instance, the training allowed clinicians to focus and “dive into each of the CAPs” to “have a good understanding” and help identify what “interventions can assist with those different issues” (Participant 9), while using a trauma-informed perspective. An improved understanding of best practice assisted clinicians in the application of the evidence-based care plans with both children/youth and parents, where having “a better understanding of the language (…) and CAPs” (Participant 4) assisted in the identification of what was “more helpful for parents compared to the youth” (Participant 8).

## Discussion

Findings suggested that the interRAI TIC training for all training groups (total, hybrid, and online groups) enhanced the use of TIC by service providers (e.g., clinicians, therapists, social workers) within mental health agencies in Ontario. Alongside this, qualitative findings suggest that clinicians felt the TIC training enhanced and validated their application of TIC with clients, helped integrate TIC into their agency, empowered their advocacy for timely services, and enabled a deeper understanding of the interRAI ChYMH and CAPs. The results from this study are notable since clinicians adopting a positive attitude toward TIC practices indicated a higher readiness to change to support TIC with their clients (Baker et al., 2016; Marvin & Volino Robinson, 2018).

When investigating a pre-post outcome for using paired sample t-tests on the ARTIC-45 questionnaire, for the total sample and online and hybrid subsamples, the *Underlying Causes of Problem Behavior and Symptoms* and *Responses to Problem Behaviour and Symptoms* subscales highlighted the changes in beliefs toward providing TIC in their clinical practice. After the interRAI TIC training, clinicians shifted their belief to reflect that the traits of their clients are more malleable and external. Focus group participants reported a more comprehensive understanding of the interRAI ChYMH and CAPs, as well as interventions for specific issues related to trauma. These evidence-informed care planning protocols provide evidence-informed supports within the context of mental health care.

In terms of the subscale, *Responses to Problem Behaviour and Symptoms*, clinicians in each sample/subsample (total, hybrid, online) shifted their beliefs to focus more heavily on safety, flexibility, and healthy relationships when working with clients who have experienced domestic violence and abuse. Key features in the training addressed attachment as well as interpersonal relations with the intent to provide intervention approaches to develop safe and secure relationships between children/youth and caregivers. Also, the interRAI TIC training included specific modules related to attachment and the importance of emotionally corrective, reparative experiences with a consistent, predictable, and emotionally attuned caregiver. Based on reports from focus group participants, their increased sense of confidence and knowledge regarding TIC concepts, including better communication and use of TIC language when working with children, youth, and families could also explain the changes in clinicians’ beliefs toward providing TIC in their practice. The importance of these changes in belief are explained by previous research which has shown that relationally integrating attachment theories to the treatment of trauma can be particularly effective (Pearlman & Courtois, 2005). Despite the evidence to support the integration of an attachment lenses in trauma interventions, research has shown that clinicians have difficulty forming therapeutic relationships with individuals who have a history of trauma (Pearlman & Courtois, 2005). The content within the interRAI TIC training, which covered the importance of a healthy therapeutic relationship and the practical ways to develop and maintain one, led to positive changes in beliefs of client’s behaviours and symptoms. Clinicians noted during the focus groups the relevance of the TIC training modules when working with clients on developing safe and healthy relationships. They reported applying the content and learning from the TIC training to work with caregiver and develop their understanding of safe and secure parent-child relationships.

In terms of the *On-the-job Behavior* subscale, clinicians in the total sample and online and hybrid subsamples shifted to a more empathetic approach to behaviours for clients who have been exposed to traumatic events, including domestic violence and abuse. The interRAI TIC training modules incorporated strategies for clinicians to help children/youth have increased empathy, to organize their feelings, and to have greater self-esteem. Also, based on feedback obtained during the focus groups, clinicians appreciated learning more about the evidence-informed research to support trauma-informed practices, including best practice when dealing with sleep disturbances in traumatized children, youth and families. By having increased knowledge of how trauma can disrupt sleep routines, which can consequently impact behavior, clinicians gained a more empathetic approach to their clients’ behaviours. Also, many of the CAPS within the training program utilize approaches that incorporate evidence-informed practice that fosters compassion and empathy to address a variety of clinical issues (e.g., behaviours such as hazardous fire involvement, harm to others, gambling, and tobacco and nicotine use). Empathy can be defined as understanding another person’s experience or expressions (Elliott et al., 2018) and research suggests empathy is a key mechanism of therapeutic change (e.g., Elliott et al., 2018; Watson et al., 2014). One study found that 30% of therapeutic outcomes are attributed to factors such as the therapeutic relationship, which includes warmth, congruence, and empathy, regardless of the therapeutic modality that is employed (Lambert & Barley, 2001). Although empathy can be an innate ability, training provides an opportunity to learn about other people’s experiences and perspectives (Gerace et al., 2015). Therefore, aligned with the TIC approach, the interRAI training serves as an extension of this empathy training by focusing on experiences that are specific to symptoms of trauma exposure, which lead to improvements on the On-the-job Behaviour Subscale.

In terms of the *Self-Efficacy at Work* subscale, the total sample and online subsample highlighted significant changes in their self-efficacy in terms of being able to meet the needs of a traumatized client. The TIC training was developed with the latest, evidence-informed practice from international experts to support clinical treatment planning. Each of the 30 CAPs provided a strong research component embedded within the modules to support clinicians’ application of TIC with child, youth, and their caregivers. interRAI TIC training provided the latest and up-to-date knowledge on the research and evidence in the field. These CAPs provide the clinician with needed best practice at the point of care. Providing the evidence-based modules enhanced clinician knowledge and experience through concrete and thorough training – providing them with increased confidence to proceed with implementing TIC strategies with their clients. Also, as reported in the focus groups, the interRAI TIC training modules supported clinicians’ improved sense of client-focused, TIC language as the training provided relevant and easy-to-understand TIC definitions, terms, and concepts for interactions with their clients. Consequently, the training may help clinicians to guide their conversations with clients using language from the modules that is easily understood and empathetic while discussing topics around possible interventions. Focus group participants also noted that the interRAI TIC training modules enhanced the communication and understanding of TIC knowledge and practices amongst their clinical teams to improve the application of TIC with clients. In addition, it provided specific trauma-informed based strategies for implementation and focus group participants noted they gained additional tools and knowledge to communicate with their clients.

For the *Reactions to Work* subscale, only the online subsample resulted in a significant improvement to the participants beliefs around the appreciation for the impact that trauma may have, and the coping strategy used. Many clinicians in the focus groups indicated they were already familiar with trauma-informed knowledge and related applications because of their educational training, as well as opportunities for further learning and experience within the field or their agency. A survey conducted in 2017 found that one in five professional psychology graduate programs in North America provide a trauma psychology course and specified practicum working with traumatized populations (Cook et al., 2017). Further, researchers suggest the critical need to integrate trauma training into psychology graduate programming (Cook et al., 2019). As such, there is a high possibility that the clinicians in our sample have received a strong basis of appreciation with consistent opportunities to continue understanding the impact of trauma in their clinical work, thus explaining the lack of significant change for two out of the three of the sample groups. In contrast, the exclusively online group demonstrated a significant improvement in appreciating the effects of vicarious traumatization and seeking support to cope with this experience. This could have been the result of the COVID-19 pandemic initiating the shift from in-person to virtual children’s mental health services (Danseco et al., 2021). Since staff engagement and receiving leadership support and training opportunities was imperative to implementing virtual care during the pandemic (Danseco et al., 2021), clinicians may have experienced an increased understanding of the importance of vicarious trauma and seeking support from colleagues and supervisors while providing virtual care. Furthermore, the pandemic and virtual services shifted the landscape and mentality of children’s mental health services (Berardini et al., 2021), where clinicians may have felt more justified to receive support for the impacts of their work. Another explanation for these findings could also be the result of the increased relevance and discussion surrounding the traumatizing effects of the COVID-19 pandemic on individual mental health, wellbeing, and stress (Bridgland et al., 2021), potentially destigmatizing trauma and mental health. As a result, clinicians in the online group may have felt more comfortable to appreciate, discuss, and get support for vicarious trauma experiences in their clinical work.

Findings from the supplementary subscale, *Personal Support of TIC* showed a lack of significant improvement for the total sample and hybrid subsample. This may be consistent with the findings from the *Reactions to Work* subscale where clinicians were already supporting the implementation of TIC prior to joining the research so their beliefs did not shift significantly. Prior to engaging in the interRAI TIC training, clinicians may have already held an appreciation for the impact of trauma on their clients, considering that clinicians may have already experienced previous trauma training, especially more novice clinicians to the field who took educational programs that integrated trauma-informed practices into the curriculum. These speculations are supported by the focus group participants who found the training to be relevant based on their individual previous educational or training knowledge and client experience with TIC. Nonetheless, participants indicated the training was an important refresher and reminder of the reasoning behind trauma-informed client practice.

The last supplementary subscale, the *System-Wide Support for TIC*, showed a lack of significant improvement for both the total sample and hybrid subsample, which may in part be explained by the lack of focus within interRAI TIC Training on system wide organizational structures. The content of the training was specifically aimed at improving outcomes for clients rather than structural change within the organization to support TIC. Nonetheless, some focus group participants highlighted the potential for the TIC training to lead to structural change within their agency. Focus group participants indicated the TIC training helped integrate trauma-informed practices into the fabric of the agency, where management and administration personnel were more understanding of the processes. Clinicians expressed the potential for the TIC training to lead to overall increased support for trauma informed practices and improvements in the implementation of TIC practices and the overall quality and timeliness of services provided by the agency. Clinicians in the focus groups also highlighted their enhanced team approach to trauma-informed care and practices, where clinical teams have a better understanding of the TIC language for communication during meetings and reports. Future research may hope to see quantitative improvements in system-wide support where organizations change their infrastructure to integrate trauma-informed care into the foundation of their organizations (e.g., Sanctuary Model; Esaki et al., 2013). Integrating additional system-wide evidence-based approaches within the interRAI TIC training would thereby help to address agency infrastructures and build TIC strategies within mental health facilities across the institution/organization.

### Impact of COVID-19 on Findings

It is possible that the COVID-19 pandemic may have influenced the online group to show a significant improvement to their self-efficacy because of the change to the delivery of client services (e.g., in-person to virtual care), as well as the clients being seen. Recent research conducted throughout the COVID-19 pandemic has indicated an increase in childhood trauma incidences (e.g., Kuehn, 2020; Kovler et al., 2021; Sserwanja et al., 2021; Usher et al., 2020; Zhang, 2022). Increased trauma may be the result of a myriad of factors, such as stay at home and lockdown government policies leading to increased social isolation for children and youth (Caron et al., 2020; Martins-Filho et al., 2020). This lends itself to problematic provision of care and intervention for these children because the COVID-19 pandemic has also resulted in a lack of screening from child welfare services and overall reduced social care that monitors and responds to child abuse and maltreatment (Caron et al., 2020; Martins-Filho et al., 2020; Rengasamy et al., 2022; Usher et al., 2020; Whaling et al., 2023; Zhang, 2022). Perhaps clientele seeking mental health services during the COVID-19 pandemic, that are using virtual services, are distinctly different from those who are seen in person (e.g., greater trauma experiences, higher socioeconomic status; Stewart et al., 2021b, 2022a, b; Stewart et al., 2023). It is possible that these lower rates of trauma within the clientele being served by mental health clinicians, is leading to increased sense of self-efficacy for clinicians because they are not working with highly traumatized clientele. Future research should investigate differences between the clientele profiles of those who have engaged in virtual versus in-person therapy.

Although the hybrid group demonstrated a positive shift in their self-efficacy to meet the needs of a traumatized client, the shift was not large enough to be of significance. Attitudes towards online therapy have exponentially shifted to be more accepting and positive since the COVID-19 pandemic (Hanley, 2021). Research indicates that the method in which a client receives therapy, either virtual or face-to-face, is accompanied by specific advantages and disadvantages (Cook & Doyle, 2002; Stoll et al., 2020). More generalized acceptance of online therapy may be the result of the specific advantages that online therapy can provide, such as increased accessibility, convenience, and specific economic advantages (Barnett & Scheet, 2003; Chester & Glass, 2006; Stoll et al., 2020). However, the disadvantages, including reduced therapist competence in providing online therapy (Harris & Birnbaum, 2015; Stoll et al., 2020) and issues forming a strong therapeutic relationship (Poh et al., 2013; Stoll et al., 2020), may result in deflated general self-efficacy to provide counselling to clientele. Thus, the lowered clinician self-efficacy to meet the needs of traumatized clientele, may be reflected within an online format, particularly when clinicians had in-person sessions to compare to.

### Clinical Implications

This study has several implications for policy, organizations implementing TIC, research, and clinical practice. A strength of this study is that both the qualitative and quantitative findings support the generation of new questions for further areas of research and have strong clinical implications.

In response to a call for action for children’s mental health services to have better systems integration, shared language and assessment tools, trauma-informed initiatives, and processes that support the efficient allocation of resources, interRAI developed the child and youth suite of mental health assessment instruments. The interRAI ChYMH, utilized in the current study, is well aligned with the United Nations rights-based model to provide trauma-informed care. Trauma-informed care within agencies is not only the implementation of TIC training, but also evidence-based assessment tools and pathways to care (Bargeman et al., 2021; United Nations General Assembly, 1989). Alongside the implementation of the interRAI TIC training, is the interRAI ChYMH, a highly evidence-based assessment tool that can support agencies through care planning and resource allocation processes (Stewart et al., 2015a, b, 2022a, b; Stewart & Hamza, 2017). Furthermore, both the TIC training and assessment tool integration into mental health agencies can support the rights-based model to providing trauma-informed care. Having assessment tools that have a common language around trauma and mental health is integral to supporting children involved with multiple clinicians or systems of care (Bargeman et al., 2021; United Nations General Assembly, 1989). The interRAI ChYMH and TIC training support the use of shared language where several participants from our study appreciated the common TIC language they received from the training. The rights-based model to TIC also includes initiatives to help prevent child maltreatment, incorporating children’s views and opinions on treatment, and incorporating an intersectional lens (Bargeman et al., 2021; United Nations General Assembly, 1989). It is suggested that further research and clinical initiatives can help to address these components of trauma-informed care.

Overall, findings demonstrate the importance of training clinicians in comprehensive and thorough TIC approach to support their practice. Findings revealed TIC training can contribute to changing clinicians’ attitudes and beliefs towards TIC. Specifically, interRAI TIC training may improve client care by shifting clinicians’ beliefs and practices to be more trauma informed and empathetic. These shifts have the potential to improve their effectiveness in meeting the treatment needs of their clients, especially those who have experienced domestic violence and abuse. As a result, there are implications for clinicians and mental health organizations to engage and invest in effective TIC training programs to support TIC implementation. TIC can be a valuable clinical approach to create an emotionally safe and respectful space for children, youth, and families to discuss and manage their concerns (Forkey et al., 2021). Moreover, it has also demonstrated improved client engagement, clinical care outcomes. practitioner well-being, and reduced service utilization (Menschner & Maul, 2016).

Findings from our study also revealed the interRAI TIC training to be effective for clinicians with different levels of education, years of experience, and discipline. Even though clinicians may be knowledgeable in TIC practices, the interRAI TIC training provided an opportunity to be refreshed on TIC, receive current and new evidence in the field, and obtain validation for their current practices. As a result, the interRAI TIC training has the potential to be applicable and effective amongst a wide range of clinicians at various mental health agencies. Furthermore, the interRAI TIC training provided clinicians with a universal, standardized approach to client care, providing an opportunity for more efficient and effective care planning and communication approach with colleagues when discussing client care. Overall, findings support the use of interRAI TIC training to develop clinicians’ attitudes and approaches to TIC with clients and amongst staff.

### Limitations and Future Directions

This study is not without limitations. Findings from the study may be limited because of the sample size. Further, the statistical power may be limited because of sample sizes differing significantly across groups. The smaller sample size in the online training group and participants being from one Canadian province (Ontario) may result in findings not representing the larger population. Future research should include a larger sample of online training participants to ensure that the current results are replicable and generalizable. Unfortunately, due to the research design we are unable to make causal conclusions on the effectiveness of the interRAI TIC training. Furthermore, it is possible that one of the outcomes identified in our research, increased system-level support for trauma-informed care, could in fact be a confounding variable that impacted the outcome of the research rather than being an outcome. Future research should take steps to control for system-level support for TIC potentially influencing research outcomes.

Regarding the qualitative findings, it is possible that selection bias was present since those who participated in the focus groups volunteered and as a result may have been more passionate to share their feedback with the research team. Furthermore, participants who may have been passionate about trauma-informed care may have had additional TIC training possibly from their education or additional certifications. As a result, these participants may have been more comfortable and well-versed discussing the implementation of the interRAI TIC training. Some participants may have felt nervous or unsure to speak truthfully and honestly in a group setting with their colleagues regarding their thoughts on the training, potentially limiting the findings. There is a possibility that participants were involved in additional trauma training and experiences during the study period that were outside the controlled study environment. As a result, these experiences could potentially influence the participants’ attitudes towards TIC. Future studies could account for any additional TIC training and experiences that participants have throughout the duration of the study.

Another limitation of the current study is the lack of follow-up to understand if the changes in TIC attitudes and practices were sustained and future research should investigate follow-up outcomes of the interRAI TIC training. Furthermore, the ARTIC and focus groups rely on clinician reports rather than observable changes or client outcomes. Future publications will highlight client-related outcomes from the interRAI TIC training. Finally, the online training (vs. hybrid training) was associated with more positive scores on some of the ARTIC subscales and should be further investigated in follow-up research to understand if there were any external factors that played a role and potentially impacting training implementation. Despite these limitations, findings from this study contribute to our understanding of how the interRAI TIC training might be influencing clinician attitudes and use of TIC practices.

The research team currently has plans to broaden the geographical regions in which the interRAI TIC training is implemented. To implement the TIC training modules, it is first necessary that the ChYMH is implemented. There is increasing uptake and interest in the ChYMH where several provinces across Canada (Prince Edward Island, Newfoundland and Labrador), with many jurisdictions mandating one or more of the interRAI instruments to support vulnerable children and their families and other Provinces have demonstrated interest in scaling up this project (British Columbia, Manitoba, Quebec), respective to their provincial needs. We plan to continue broadening the implementation of TIC with more agencies across Canada with adjustments to our training program based on the evaluation results. Attention to system organizational changes and specific barriers to implementation (e.g., competing priorities, clinician time constraints, resources, inter-agency collaboration, policy and procedures; Huo et al., 2023), will be needed to enhance the uptake of TIC, identifying when TIC training works, and under what conditions. Furthermore, reports suggest key factors necessary for successfully implementing trauma-informed care in mental health services. Key ingredients of trauma-informed practices and organizations include leading and communicating about the process, engaging patients in planning the organization, training clinical and non-clinical staff in TIC, fostering a safe environment, and preventing secondary trauma in staff (Menschner & Maul, 2016). In a treatment protocol by the Substance Abuse and Mental Health Services Administration (2014) it is required that TIC has organizational commitment, cultural changes, and heavy involvement from the implementing agency.

## Conclusion

The study used a mixed-method approach to understand clinicians’ beliefs about TIC and the interRAI TIC training program. A pre-post evaluation was used to capture changes in clinicians’ beliefs about TIC upon receiving interRAI’s evidence-informed TIC training. Thematic analysis was used to supplement the findings by gaining a deeper understanding of clinician’s perspective of the training program. It was revealed that overall, each training group within this study had more favourable attitudes towards TIC after receiving interRAI TIC training. Additionally, clinicians indicated that the training supported their use of TIC, helped with TIC implementation in their agencies, and encouraged advocacy for providing timely services to clients. This study has implications for mental health agencies to include TIC training within their organization to support all clientele, and especially those with a history of trauma exposure. Furthermore, the current study demonstrated that the interRAI TIC training has the potential to support clinician’s practice to be more trauma-informed for children, youth and their caregivers who have experienced domestic violence and abuse.

## Appendix A

### interRAI Child and Youth Mental Health Collaborative Action Plans

1. Attachment CAP.
2. Caffeine Use CAP.
3. Caregiver Distress CAP.
4. Communication CAP.
5. Control Interventions CAP.
6. Criminality Prevention CAP.
7. Education CAP.
8. Gambling CAP.
9. Harm to Others CAP.
10. Hazardous Fire Involvement CAP.
11. Informal Support CAP.
12. Interpersonal Conflict CAP.
13. Life Skills CAP.
14. Medication Adherence CAP.
15. Medication Review CAP.
16. Parenting CAP.
17. Physical Activity CAP.
18. Readmission CAP.
19. Sexual Behaviour CAP.
20. Sleep Disturbance CAP.
21. Social and Peer Relationships CAP.
22. Strengths CAP.
23. Substance Use CAP.
24. Suicidality and Purposeful Self Harm CAP.
25. Support Systems for Discharge CAP.
26. Tobacco and Nicotine Use CAP.
27. Transitions CAP.
28. Traumatic Life Events CAP.
29. Video Gaming CAP.
30. Weight Management CAP.

## Appendix B

### Focus Group Questions – Training Component

1. Do you have an improved trauma-based mental health literacy as a result of completing the interRAI TIC project training?
2. To what degree, if at all, do you feel that your enhanced knowledge of trauma-informed care will help to improve the early identification of problems associated with domestic violence and abuse?
3. To what degree, if at all, do you feel more confident to apply TIC with children/youth after you received the training?
4. As a result of the interRAI TIC training, do you feel more confident to engage in discussions with youth and their guardians around TIC?
5. Do you notice a change in the way you communicate to one another about the needs of children/youth after completing the TIC training linked to the interRAI CHYMH and CAPS?
6. To what degree, if at all, do you feel the learning from the educational modules will help to improve your understanding and practice of trauma-informed care to the application of the interRAI ChYMH CAPS for children and youth exposed to domestic violence and abuse?
7. As a result of the training, did you experience an increased interest in the interRAI ChYMH and utility of the CAPs?
8. As a result of the interRAI TIC training, have children/youth clients who have experience DVA been referred to other services in a timelier manner? *(Not asked for one agency as they recently completed the training)*
9. Do you have any comments you would like to discuss related to the interRAI TIC project?
10. Anything we could have changed to make your TIC learning experience better? (i.e. Content, delivery).
